# Supplementary material for: Long-term imaging of dorsal root ganglia in awake behaving mice
Source: Nat Commun. 2019 Jul 12;10:3087. doi: 10.1038/s41467-019-11158-0 (PMC6625980; doi:10.1038/s41467-019-11158-0)
Supplement: Supplementary file 1 — Supplementary Information [file 41467_2019_11158_MOESM1_ESM.pdf]

Supplementary Information

Long-term imaging of dorsal root ganglia in awake behaving mice

Chen et al.

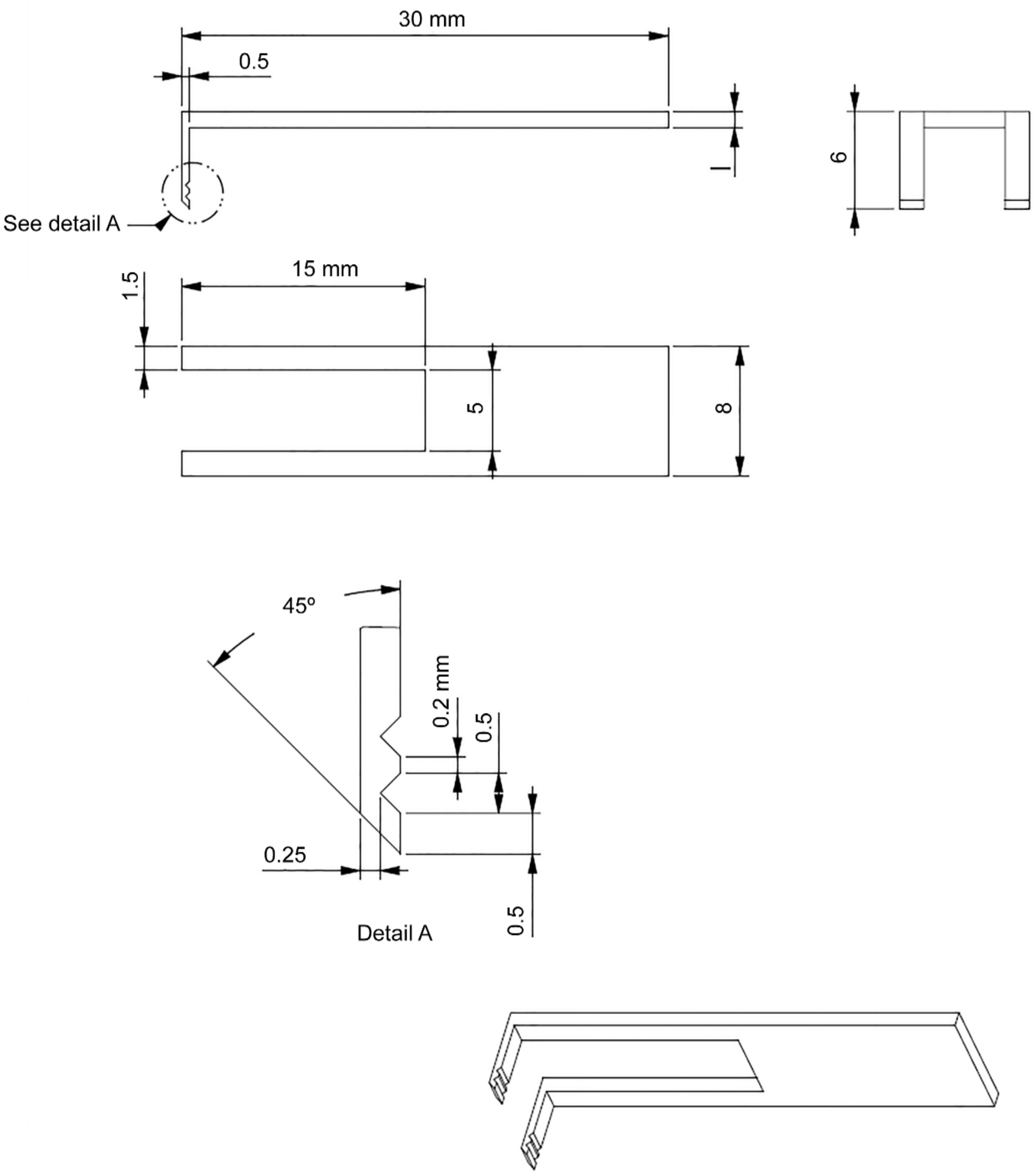

Scale 5:1

Part: L-shaped long plate
Quantity: 1
Material: 316 stainless steel

Supplementary Figure 1| Mechanical drawing of the long plate.

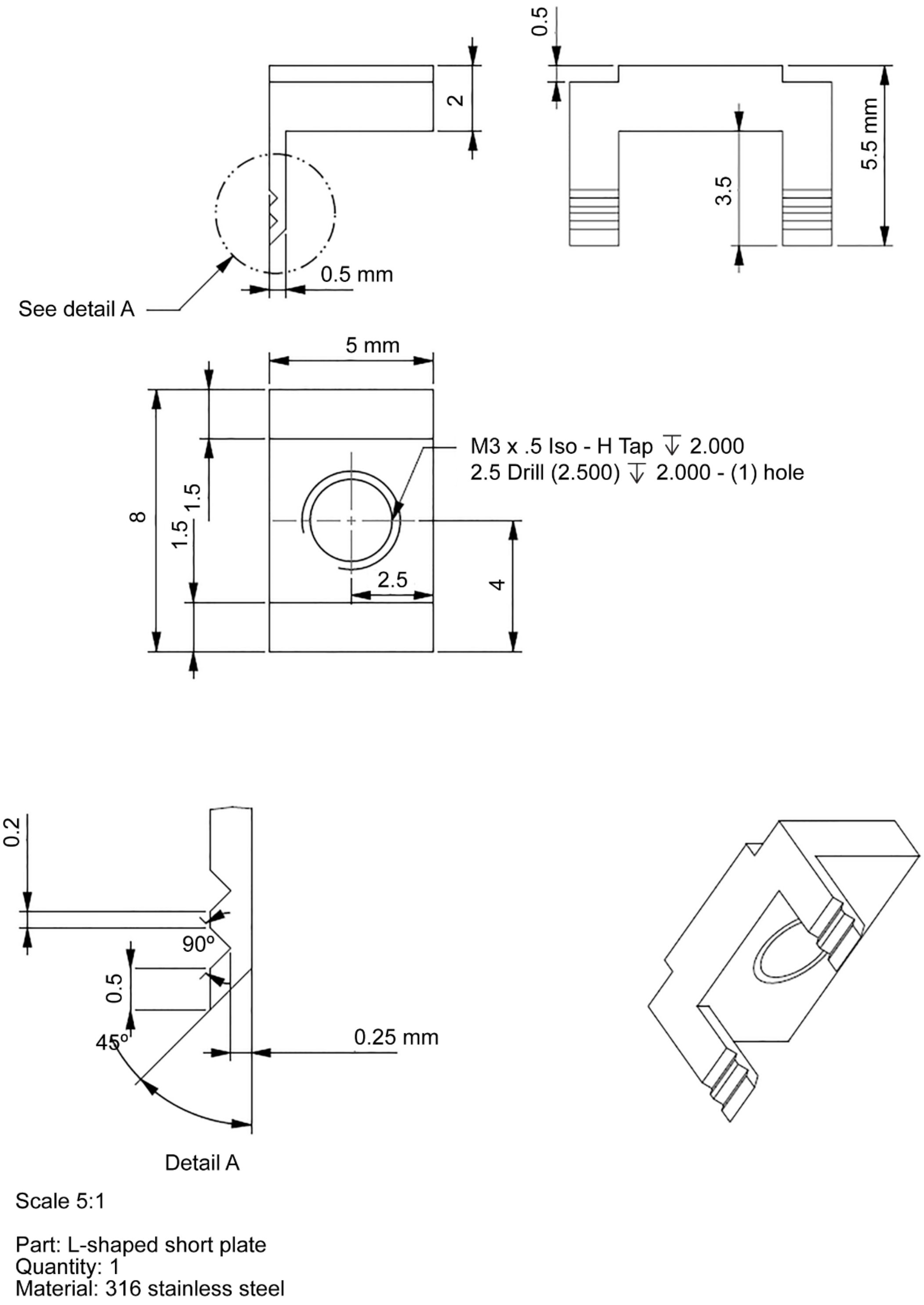

Supplementary Figure 2| Mechanical drawing of the short plate.

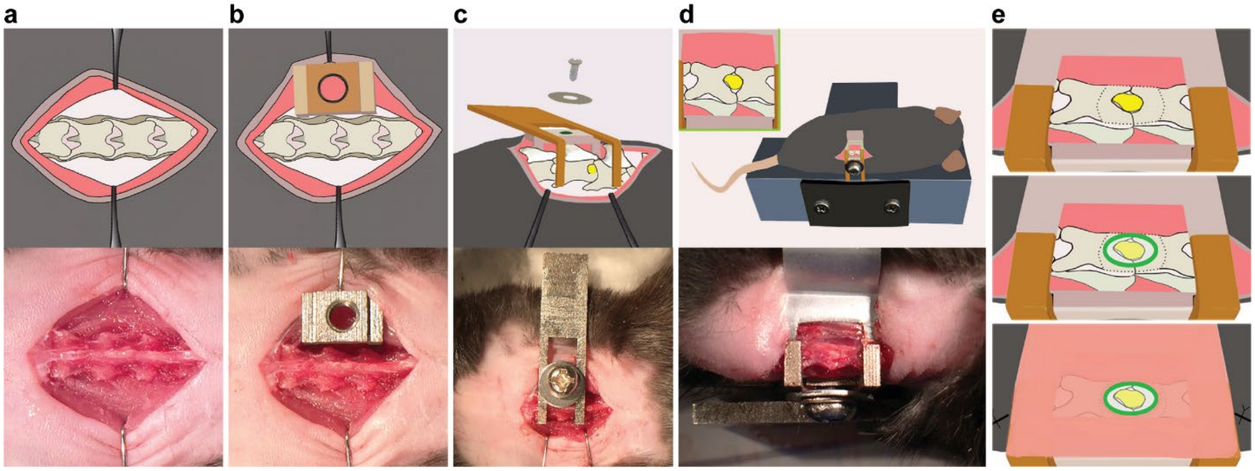

Supplementary Figure 3| Surgical procedures for vertebral window implantation. (**a-b**) The exposed vertebrae (L4-L5) were clamped by two L-shaped plates centering on the articular processes between L4 and L5 after muscle had been retracted. (**c**) Mounting plates were locked with a screw. (**d**) The mouse with attached mounting plates was placed lying on its right side, and the arm of the long plate was plugged and secured to a heavy metal base. After the muscle tissue had been held back with a thin aluminum sheet, L4 DRG was exposed. (**e**) The raised part of articular processes around L4 DRG was gently removed with the high-speed micro-drill, leaving the surface of the trimmed bone and DRG in the same optical plane (dotted line part). DRG was sealed with a 2-mm diameter cover glass after applying a thin layer of silicone elastomer to the surface of DRG. Cyanoacrylate glue and dental acrylic were used to fix the cover glass to the mounting plates. An aluminum ring (green) edged dental acrylic, serving as the frame of DRG window.

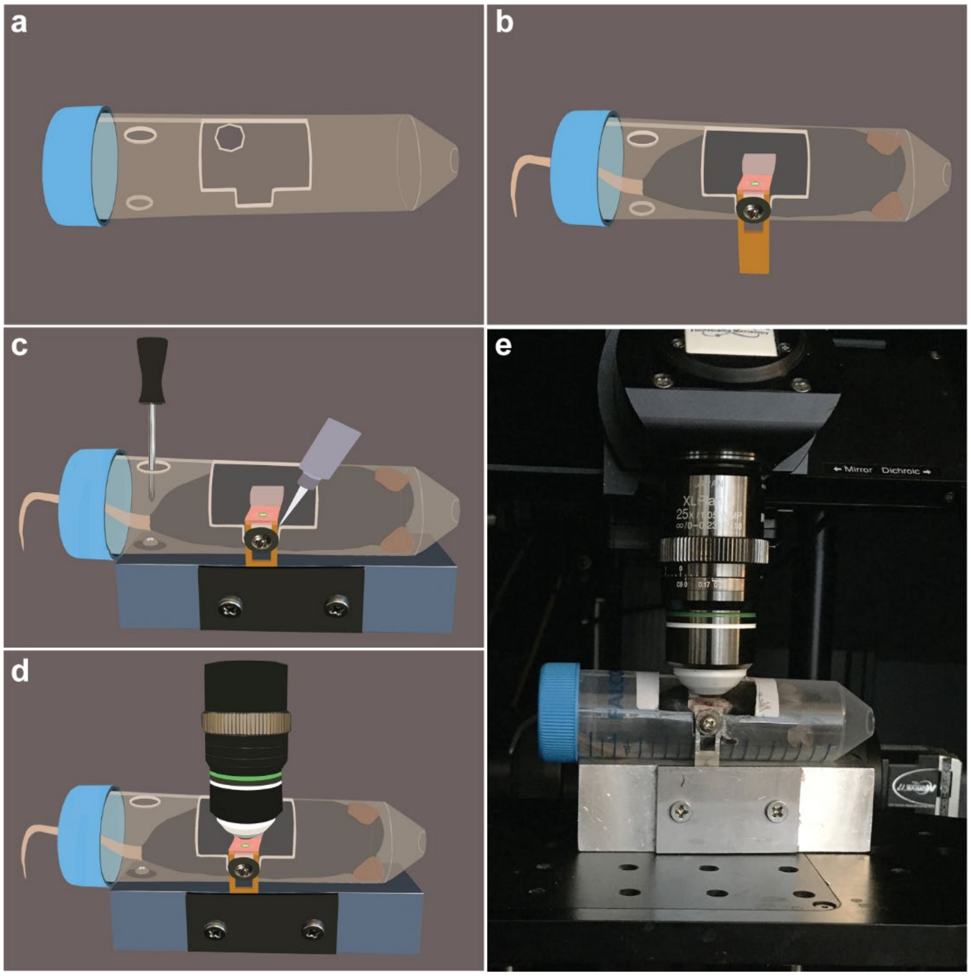

Supplementary Figure 4| Procedure to prepare DRG imaging in awake, vertebrae-restrained animals. (**a-b**) To minimize motion-induced artifacts during imaging, the mouse was placed in a 2.9-cm-diameter plastic tube with a custom-made window allowing access to the exposed DRG for imaging. (**c**) The vertebral mount was secured to the metal base with a screw. Cyanoacrylate glue may be applied to the mounting plates to improve the stability of the device. (**d, e**) The mouse together with the metal base was placed under the microscope, ready for imaging.
