# Supplementary material for: Long-term imaging of dorsal root ganglia in awake behaving mice
Source: Nat Commun. 2019 Jul 12;10:3087. doi: 10.1038/s41467-019-11158-0 (PMC6625980; doi:10.1038/s41467-019-11158-0)
Supplement: Supplementary file 3 — Description of Additional Supplementary Files [file 41467_2019_11158_MOESM3_ESM.pdf]

Description of Additional Supplementary Files

File name: Supplementary Movie 1

Description: Ca^{2+} imaging of DRG neurons expressing GCaMP6s during awake or anesthesia conditions.

File name: Supplementary Movie 2

Description: Repeated Ca^{2+} imaging of DRG neurons in an awake mouse over the course of 21 days.

File name: Supplementary Movie 3

Description: Ca^{2+} imaging of DRG neurons in an awake mouse before and after plantar saline injection.

File name: Supplementary Movie 4

Description: Ca^{2+} imaging of DRG neurons in an awake mouse before and after plantar formalin injection.
